# Non-Target Effects of *Trichoderma* Spore Suspensions and Secondary Metabolites on Phytoseiid Predatory Mites

**DOI:** 10.3390/jof12060382

**Published:** 2026-05-23

**Authors:** Cihan Aslı, Yunus Korkom, Daniel Carrillo, Ibrahim Cakmak

**Affiliations:** 1 Graduate School of Natural and Applied Sciences, Aydin Adnan Menderes University, 09010 Aydin, Türkiye; cihanasli1071@gmail.com; 2 Department of Plant Protection, Faculty of Agriculture, Aydin Adnan Menderes University, 09010 Aydin, Türkiye; yunus.korkom@adu.edu.tr; 3 Tropical Research and Education Center, Institute of Food and Agricultural Sciences, University of Florida, Homestead, FL 33031, USA; dancar@ufl.edu

**Keywords:** *Amblyseius swirskii*, *Neoseiulus californicus*, *Phytoseiulus persimilis*, *Trichoderma afroharzianum*, *Trichoderma virens*, biological control, non-target effects, mycoparasitic fungi

## Abstract

Fungi of the genus *Trichoderma* have attracted attention because of their potential activity against phytophagous mites; however, information regarding their non-target effects on predatory mites remains limited. This study evaluated the effects of spore suspensions and secondary metabolites of *Trichoderma afroharzianum* Tr132 and *Trichoderma virens* Tvr2 on three predatory mite species widely used in biological control programs: *Phytoseiulus persimilis*, *Neoseiulus californicus*, and *Amblyseius swirskii*. Predator egg hatchability and adult mortality were assessed under laboratory conditions. Spore suspension treatments did not significantly affect egg hatchability, which remained high (97–99%) across all predator species. In contrast, secondary metabolites slightly reduced egg hatchability to 94–96%, compared with 99.5% in the control. Exposure to spore suspensions caused moderate mortality in adult predatory mites, increasing from 10 to 13% at 3 days post-application (dpa) to 15–19% at 6 dpa. Secondary metabolites produced higher mortality that increased over time, reaching 9–11% at 1 dpa, 17–18% at 3 dpa, and up to 22–25% at 6 dpa. Mortality responses were similar among predator species. Overall, *Trichoderma* applications had minimal effects on predator egg hatchability but caused moderate mortality in adult predatory mites, particularly following exposure to secondary metabolites. These findings highlight the importance of evaluating the compatibility of *Trichoderma*-based products with beneficial predatory mites before their integration into IPM programs.

## 1. Introduction

The two-spotted spider mite, *Tetranychus urticae* Koch (Acari: Tetranychidae), is one of the most destructive and widely distributed phytophagous mites worldwide. This highly polyphagous species has been recorded on more than 1200 host plants, including vegetables, fruit trees, ornamental plants, and industrial crops [1]. Under favorable environmental conditions, *T. urticae* can produce multiple generations and rapidly increase its population density. Feeding by this mite damages mesophyll cells, resulting in chlorotic and necrotic spots, leaf deformation, and reduced photosynthetic capacity. Feeding on flowers and fruits may also cause flower wilting and fruit discoloration, leading to yield losses of up to 80% in heavily infested crops [2,3].

Management of *T. urticae* has traditionally relied on chemical acaricides. However, extensive pesticide use has created several challenges, including environmental contamination, negative effects on non-target organisms, and the rapid development of acaricide resistance. Consequently, increasing attention has been directed toward alternative and sustainable pest management strategies. Among these, biological control using predatory mites of the family Phytoseiidae represents one of the most effective approaches for suppressing spider mite populations in both greenhouse and field crops. Predatory mites possess several advantageous traits, including high reproductive capacity, strong foraging ability, rapid dispersal, adaptability to diverse environmental conditions, and a high degree of prey specificity [4]. In particular, *Phytoseiulus persimilis* Athias-Henriot, *Neoseiulus californicus* (McGregor)*,* and *Amblyseius swirskii* Athias-Henriot are widely used and commercially available biological control agents that play a major role in integrated pest management (IPM) programs [5,6,7].

Microbial biological control agents have also been extensively investigated as complementary tools for spider mite management. Entomopathogenic fungi and their secondary metabolites are of particular interest because of their ability to infect or negatively affect arthropod pests [8,9,10,11]. Among these, fungal genera such as *Metarhizium*, *Beauveria*, and *Lecanicillium* have been widely studied for their pathogenicity against spider mites. For example, Chouikhi et al. [10] reported mortality rates of 93%, 95%, and 98% in eggs, immature stages, and adults of *T. urticae*, respectively, following treatment with *Beauveria bassiana* and *Lecanicillium muscarium*. However, microbial agents may also harm beneficial arthropods. Dogan et al. [12] demonstrated that spore suspensions of *Metarhizium brunneum* caused mortality rates ranging from 57.5 to 99.5% in *Phytoseiulus persimilis* and 51.5–90.5% in *Neoseiulus californicus*, emphasizing the importance of evaluating potential non-target effects.

Recently, species of the genus *Trichoderma* have attracted increasing attention because of their diverse biological activities. These fungi are widely known for their antagonistic effects against plant pathogens, plant growth-promoting properties, and their ability to induce plant defense responses [13,14,15]. In addition to their mycoparasitic activity against plant pathogens, *Trichoderma* spp. have also been reported to reduce infestations of several arthropod pests and are increasingly incorporated into sustainable crop protection programs [16]. More recent studies have explored the potential of *Trichoderma* species, their spore suspensions, and secondary metabolites against spider mites [11,17,18,19,20,21]. These studies demonstrated that *Trichoderma* treatments can cause substantial mortality in different developmental stages of *T. urticae*, particularly in motile stages, highlighting their potential as biological control agents.

Despite these promising results, the ecological compatibility between *Trichoderma*-based applications and beneficial arthropods remains poorly understood. In particular, studies evaluating the effects of *Trichoderma* on predatory mites are scarce. One of the few available studies reported moderate mortality in eggs, immature stages, and adults of *Phytoseiulus persimilis* exposed to *Trichoderma* spore suspensions [22]. Given the critical role of predatory mites in biological control programs, assessing potential non-target effects of *Trichoderma* applications is essential before integrating these fungi into IPM strategies.

This study evaluated the effects of spore suspensions and secondary metabolites of *Trichoderma afroharzianum* and *Trichoderma virens* on three commonly used predatory mites: *Phytoseiulus persimilis*, *Neoseiulus californicus*, and *Amblyseius swirskii*. We hypothesized that although these fungal treatments are effective against spider mites, their impact on predatory mites may vary depending on the fungal species and the type of application.

## 2. Materials and Methods

### 2.1. Plant Production and Rearing of Tetranychus urticae

Common bean plants (*Phaseolus vulgaris* var. *barbunia*) were grown for use in laboratory bioassays and for maintaining *Tetranychus urticae* colonies. Plants were cultivated in plastic pots (11 × 11 cm) containing a peat–perlite mixture (5:1, *v*/*v*) and maintained in climate-controlled chambers at 25 ± 2 °C, 60 ± 10% relative humidity (RH), and a 16:8 h light:dark (L:D) photoperiod until reaching the 5–6 leaf stage.

Colonies of *T. urticae* were established from individuals originally collected from cotton fields in Germencik (Aydın Province, Türkiye) in 2017 and have been continuously maintained on bean plants in a separate climate chamber under similar environmental conditions. Clean, mite-free plants were periodically infested with mixed developmental stages of *T. urticae* to ensure stable prey availability throughout the experimental period.

### 2.2. Predatory Mite Cultures

Three phytoseiid predatory mite species were used in this study: *Neoseiulus californicus*, *Phytoseiulus persimilis*, and *Amblyseius swirskii*. *N. californicus* individuals were collected from strawberry fields in Kuşadası (Aydın Province, Türkiye), *P. persimilis* from cotton fields in Germencik (Aydın Province), and *A. swirskii* was obtained from Çukurova University, Adana, Türkiye.

Predatory mites were reared on *T. urticae*-infested bean leaves placed on inverted plastic pots (8 × 10 cm) inside plastic containers (9.2 L) filled with water to prevent mite escape and cross-contamination. The container edges were coated with petroleum jelly as an additional barrier. Cultures were maintained at 25 ± 1 °C, 70 ± 10% RH, and a 16:8 h (L:D) photoperiod. Fresh *T. urticae*-infested bean leaves were supplied three times per week as a food source.

### 2.3. Preparation of Trichoderma Spore Suspensions

The study used *Trichoderma afroharzianum* Tr132 (GenBank accession number: OP847792), originally isolated from almond rhizosphere soil, and *Trichoderma virens* Tvr2 (GenBank accession number: MZ853844), isolated from strawberry rhizosphere soil in Aydın Province, Türkiye. These local wild isolates were obtained from a previous study [23] and maintained at −20 °C until use. Isolates were cultured on potato dextrose agar (PDA) at 25 °C for 7 days under a 12:12 h (L:D) photoperiod in an incubator (Memmert IF55, Memmert GMbH, Buchenbach, Germany). To prepare spore suspensions, the surface of each culture was flooded with sterile distilled water containing Tween 20 (0.05% *v*/*v*), and spores were gently dislodged using a sterile glass rod. The resulting suspensions were vortexed and filtered through four layers of sterile cheesecloth to remove mycelial fragments [21,24,25]. Spore concentrations were determined using a hemocytometer under a light microscope (Olympus CX21, Evident Corporation, Tokyo, Japan) and adjusted to a final concentration of 1 × 10^8^ spores mL^−1^ prior to bioassays.

### 2.4. Production of Trichoderma Secondary Metabolites

*Trichoderma afroharzianum* and *T. virens* isolates were initially grown on PDA at 25 ± 1 °C for 3 days. Two agar discs (7 mm diameter) were excised from the actively growing margins of each culture and transferred into 100 mL of sterile yeast peptone glucose (YPG) broth previously autoclaved at 121 °C for 15 min, 1 atm. YPG broth was selected because previous studies showed that secondary metabolites produced in this medium exhibited higher acaricidal activity than metabolites produced in several other culture media [11,21]. YPG liquid cultures were incubated at 25 ± 1 °C for 30 days on a 120 rpm orbital shaker to allow accumulation of *Trichoderma* extracellular secondary metabolites [11,26,27]. At the end of the incubation period, cultures were sequentially filtered through Whatman No. 4 filter paper and a 0.22 µm membrane filter (16534-K, 28 mm SFCA Minisart, Sartorius, Göttingen, Germany) to obtain cell-free culture filtrates. These filtrates were used directly in bioassays.

### 2.5. Bioassays

All bioassays were conducted in 9 cm-diameter plastic Petri dishes. Each dish contained moistened cotton (approximately 6.2 cm in diameter and 1 cm in height), and the space between the cotton and Petri dish wall was filled with water to prevent mite escape. A clean bean leaf disc (6 cm-diameter) was placed on the cotton with the abaxial surface facing upward. All arenas were prepared immediately prior to use. All treatments applied to predatory mite eggs or adults were arranged in a completely randomized design with five replicates per treatment. The experiments were repeated four times for each predatory mite species. All experiments were conducted under controlled environmental conditions (25 ± 1 °C, 70 ± 10% RH, 16:8 h L:D).

#### 2.5.1. Effects of Trichoderma Spore Suspension and Secondary Metabolite Filtrates on Egg Hatchability

Egg bioassays were conducted using 24 h-old eggs of *N. californicus*, *P. persimilis*, or *A. swirskii* obtained from the laboratory cultures. Eggs were transferred onto bean leaf discs using a fine camel-hair brush (No. 0.00), with 10 eggs placed in each Petri dish. Spore suspensions or secondary metabolite filtrates of *T. afroharzianum* or *T. virens* were applied to the leaf surface using a spray tower (Burkard, UK) calibrated to deliver 2 mL per dish at 1 atm pressure. For control treatments, sterile distilled water containing Tween 20 (0.05% *v*/*v*) was used for spore suspension assays, while sterile YPG medium served as the control for secondary metabolite assays. Egg hatchability was recorded six days post-application (dpa) under a stereomicroscope (Leica EZ4, Leica Microsystems, Wetzlar, Germany).

#### 2.5.2. Effects of Trichoderma Spore Suspension and Secondary Metabolite Filtrates on Adult Females

For adult bioassays, bean leaf discs were infested with 150–200 individuals of mixed developmental stages of *T. urticae* using a fine brush. After a 2 h acclimation period to allow prey establishment, adult female predatory mites (10 individuals per dish) were transferred onto the leaf discs. Spore suspensions or secondary metabolite filtrates were applied using the spray tower under the same conditions as described for the egg assays. To ensure continuous prey availability, additional *T. urticae* individuals (150–200 per dish) were supplied at 1 and 3 dpa. Predator mortality was recorded at 3 and 6 dpa with spore suspensions and at 1, 3, and 6 dpa with secondary metabolite filtrates. Individuals were considered dead if no movement was observed after gentle probing with a fine brush. To confirm fungal infection, dead predatory mites from spore suspension treatments were transferred onto *Trichoderma* selective medium (TSM). *Trichoderma* spp. were successfully re-isolated from mite cadavers after incubation and identified microscopically [21].

### 2.6. Statistical Analysis

The percentage of egg hatchability or adult mortality data were arcsine transformed prior to analysis to meet assumptions of normality and homogeneity of variance. Transformed data were analyzed using a Generalized Linear Model (GLM), and differences among treatment means were compared using Tukey’s multiple comparison test at a significance level of *p* < 0.05 (SPSS version 29 software).

## 3. Results

### 3.1. Effects of Trichoderma Spore Suspension and Secondary Metabolite Filtrates on Egg Hatchability

**Figure 1:**
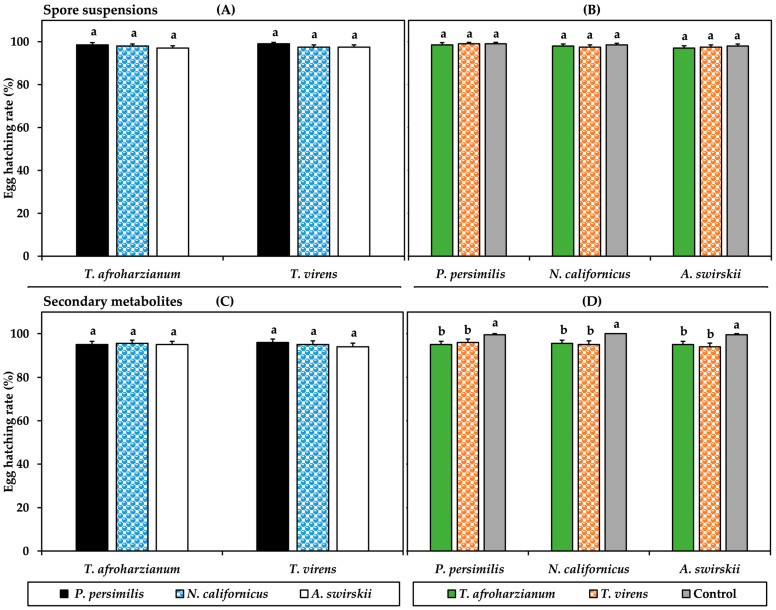
Egg hatchability rate (%) of *Phytoseiulus persimilis*, *Neoseiulus californicus*, and *Amblyseius swirskii* after exposure to spore suspensions and secondary metabolites of *Trichoderma afroharzianum* and *T. virens*. (**A**) Spore suspensions, *Trichoderma* species comparison. (**B**) Spore suspensions, treatment comparison within predatory mite species. (**C**) Secondary metabolites, *Trichoderma* species comparison. (**D**) Secondary metabolites, treatment comparison within predatory mite species. Bars represent mean values ± SE. Different letters above bars indicate significant differences according to Tukey’s test (*p* < 0.05).

Spore suspension of *T. afroharzianum* and *T. virens* did not affect egg hatchability of *P. persimilis*, *N. californicus*, and *A. swirskii* (Figure 1). Six days post-application (dpa), hatch rates ranged between 97 and 99% for all species, with no significant differences among treatments or species (*T. afroharzianum*: F = 0.901; *T. virens*: F = 0.919; all *p* > 0.05; Figure 1A). Similarly, when analyzed separately for each species, egg hatchability did not differ significantly among treatments and the control (*P. persimilis*: F = 0.027; *N. californicus*: F = 0.300; *A. swirskii*: F = 0.256, all *p* > 0.05; Figure 1B).

Secondary metabolite treatments slightly reduced egg hatchability. Hatch rates ranged from 94 to 96% in *Trichoderma* treatments compared with 99.5% in the control. Although no significant differences were observed among predator species (*T. afroharzianum*: F = 0.052; *T. virens*: F = 0.439; all *p* > 0.05; Figure 1C), significant differences between treatments and the control were detected when species were analyzed individually (*P. persimilis*: F = 3.702, *p* < 0.05; *N. californicus*: F = 4.891, *p* < 0.05; *A. swirskii*: F = 5.111, *p* < 0.01; Figure 1D).

### 3.2. Effects of Trichoderma Spore Suspension and Secondary Metabolite Filtrates on Adult Females

**Figure 2:**
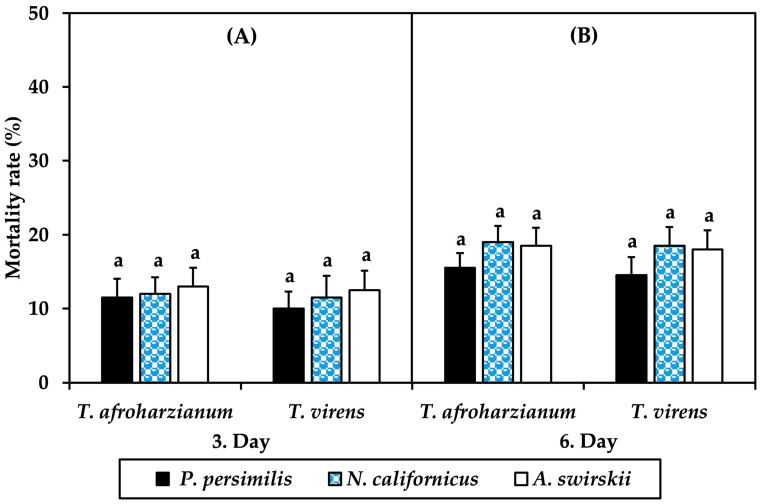
Mortality rate (%) of adult predatory mites after exposure to spore suspensions of *Trichoderma afroharzianum* and *T. virens* at (**A**) 3 and (**B**) 6 days post-application (dpa). Bars represent mean values ± SE. Different letters above bars indicate significant differences according to Tukey’s test (*p* < 0.05).

*Trichoderma* spore suspensions caused a slight increase in adult *P. persimilis, N. californicus,* and *A. swirskii* mortality over time. In the *T. afroharzianum* treatment, mortality at 3 dpa was 11.5%, 12%, and 13% for *P. persimilis*, *N. californicus*, and *A. swirskii*, respectively, increasing to 15.5%, 19%, and 18.5% at 6 dpa. Mortality did not differ significantly among the three predator species at either observation time (3 dpa: F = 0.096; 6 dpa: F = 0.752; all *p* > 0.05; Figure 2A,B). A similar pattern was observed in the *T. virens* treatment, where mortality reached 10%, 11.5%, and 12.5% at 3 dpa and increased to 14.5%, 18.5%, and 18% at 6 dpa for *P. persimilis*, *N. californicus*, and *A. swirskii*, respectively, again with no significant differences among species (3 dpa: F = 0.233; 6 dpa: F = 0.749; all *p* > 0.05; Figure 2A,B).

**Figure 3:**
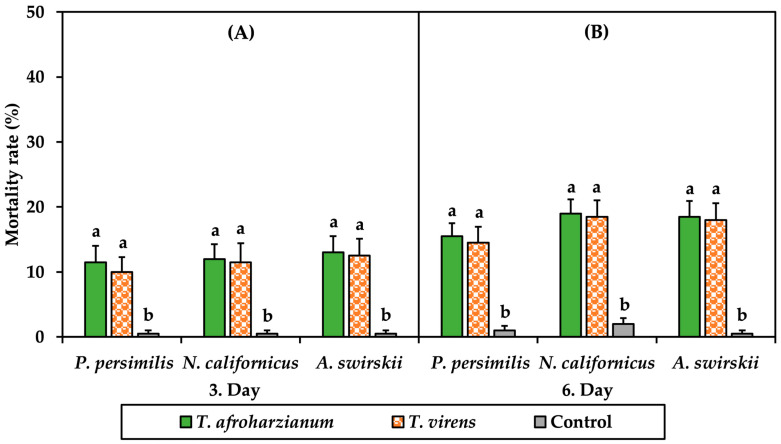
Species-specific mortality rate (%) of adult predatory mites following exposure to spore suspensions of *Trichoderma afroharzianum* and *T. virens* at (**A**) 3 and (**B**) 6 post-application (dpa). Bars represent mean values ± SE. Different letters above bars indicate significant differences according to Tukey’s test (*p* < 0.05).

When each species was analyzed separately, mortality in both *Trichoderma* treatments was significantly higher than in the control. For *P. persimilis*, mortality reached 11.5% and 10% in the *T. afroharzianum* and *T. virens* treatments, respectively, compared with 0.5% in the control at 3 dpa, increasing to 15.5% and 14.5% at 6 dpa (3 dpa: F = 8.813; 6 dpa: F = 18.671; all *p* < 0.01). Comparable trends were observed for *N. californicus* (3 dpa: F = 9.081; 6 dpa: F = 23.258; all *p* < 0.01) and *A. swirskii* (3 dpa: F = 11.161; 6 dpa: F = 24.288; all *p* < 0.01; Figure 3A,B).

**Figure 4:**
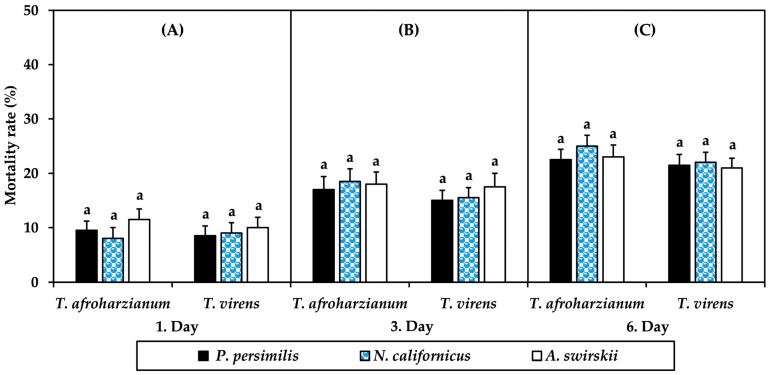
Mortality rate (%) of adult predatory mites after exposure to secondary metabolites of *Trichoderma afroharzianum* and *T. virens* at (**A**) 1, (**B**) 3 and (**C**) 6 post-application (dpa). Bars represent mean values ± SE. Different letters above bars indicate significant differences according to Tukey’s test (*p* < 0.05).

Secondary metabolites of both *Trichoderma* species increased adult predator mortality progressively over time (Figure 4). In the *T. afroharzianum* treatment, mortality in *P. persimilis*, *N. californicus*, and *A. swirskii* was 9.5%, 8%, and 11.5% at 1 dpa, rising to 17%, 18.5%, and 18% at 3 dpa and reaching 22.5%, 25%, and 23% at 6 dpa. Despite these increases, no significant differences were detected among predator species at any observation time (1 dpa: F = 1.187; 3 dpa: F = 0.125; 6 dpa: F = 0.616; all *p* > 0.05; Figure 4A–C). The *T. virens* metabolite treatment produced a comparable response, with mortality rates of 8.5%, 9%, and 10% at 1 dpa; 15%, 15.5%, and 17.5% at 3 dpa; and 21.5%, 22%, and 21% at 6 dpa for *P. persimilis*, *N. californicus*, and *A. swirskii*, respectively, again without significant interspecific differences (1 dpa: F = 0.165; 3 dpa: F = 0.421; 6 dpa: F = 0.071; all *p* > 0.05; Figure 4A–C).

**Figure 5:**
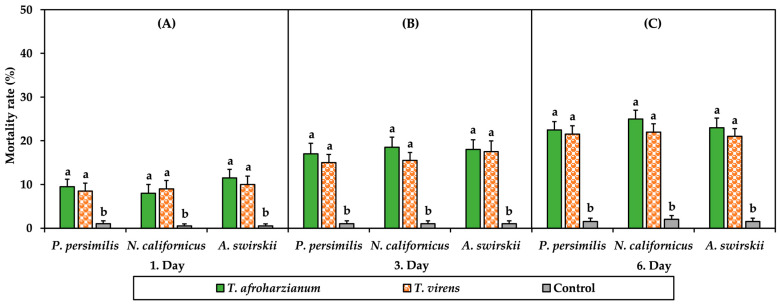
Species-specific mortality rate (%) of adult predatory mites following exposure to secondary metabolites of *Trichoderma afroharzianum* and *T. virens* at (**A**) 1, (**B**) 3 and (**C**) 6 post-application (dpa). Bars represent mean values ± SE. Different letters above bars indicate significant differences according to Tukey’s test (*p* < 0.05).

In contrast, species-level analyses revealed significantly higher mortality in both metabolite treatments compared with the control at all observation times (Figure 5). For example, mortality in *P. persimilis* increased from 9.5 to 8.5% at 1 dpa to 22.5–21.5% at 6 dpa in the *T. afroharzianum* and *T. virens* treatments (respectively), while remaining below 1.5% in the control (1 dpa: F = 9.700; 3 dpa: F = 22.951; 6 dpa: F = 50.258; all *p* < 0.01; Figure 5A–C). Similar patterns were observed for *N. californicus* (1 dpa: F = 8.175; 3 dpa: F = 27.831; 6 dpa: F = 55.328; all *p* < 0.01) and *A. swirskii* (1 dpa: F = 13.683; 3 dpa: F = 23.352; 6 dpa: F = 47.670; all *p* < 0.01; Figure 5A–C).

## 4. Discussion

The present study demonstrates that spore suspensions and secondary metabolites of *Trichoderma afroharzianum* and *T. virens* exert stage-dependent and treatment-type–dependent effects on three predatory mite species, with eggs remaining largely unaffected and adults showing moderate susceptibility. When interpreted alongside previous literature on *Trichoderma*–mite interactions and pesticide selectivity in phytoseiids, these findings provide important insights into the compatibility of fungal-based products with biological control agents.

The negligible effect of *Trichoderma* spore suspensions on predatory mite egg hatchability (≥94%) indicates that eggs represent a highly tolerant developmental stage. This finding is consistent with previous studies reporting variable but often limited ovicidal activity of *Trichoderma* spore suspensions against *T. urticae* [17,20]. Asrav et al. [21] demonstrated that *Trichoderma* spore suspensions were ineffective against *T. urticae* eggs, likely due to the structural complexity of the egg chorion, which restricts spore adhesion, germination, and enzymatic penetration. Similarly, other entomopathogenic fungi have shown relatively low efficacy against spider mite eggs [12], indicating that the chorion functions as a general physical and chemical barrier across mite species.

In contrast, *Trichoderma* secondary metabolites have been reported to exhibit moderate ovicidal activity against *T. urticae* (e.g., 54.0–57.8% mortality; [11]). However, in the present study, these metabolites did not significantly affect predatory mite eggs. This discrepancy indicates a clear difference in susceptibility between pest and beneficial species, potentially associated with differences in chorion structure or the shorter embryonic development time of phytoseiids, which reduces their effective exposure period. These findings are consistent with those of Cevizci et al. [8], who reported negligible ovicidal effects of bacterial secondary metabolites on *P. persimilis* and *N. californicus*. Collectively, the available evidence suggests that predatory mite eggs—particularly those of the family Phytoseiidae—are highly tolerant to microbial-derived compounds.

In comparison with the egg stage, adult predatory mites exhibited measurable mortality, with secondary metabolites (up to 25% mortality at 6 dpa) being more toxic than spore suspensions (~19%). The effect of spore suspensions is notably lower than the 50–65% mortality reported for *T. urticae* [21] and considerably less severe than that caused by specialized entomopathogens such as *Metarhizium brunneum*, which can induce >50% mortality in phytoseiids [12]. This relatively low pathogenicity likely reflects the ecological roles of *Trichoderma* spp. as mycoparasites and plant symbionts rather than specialized arthropod pathogens, resulting in limited adaptation to infect arthropod hosts [25,26,27,28].

The higher toxicity of secondary metabolites compared to spores indicates that the primary mode of action is chemical rather than infectious. The progressive increase in mortality over time suggests a cumulative toxic effect, likely mediated through membrane disruption and metabolic interference following both direct contact and ingestion via contaminated prey (*T. urticae*). Several secondary metabolites produced by *Trichoderma* spp., including peptaibols, trichodermin, gliotoxin, and harzianopyridone, have previously been associated with insecticidal or acaricidal activity through membrane disruption and interference with cellular metabolism [27]. Such exposure pathways are particularly relevant for phytoseiid mites due to their active foraging behavior. Despite this, mortality remained substantially lower than the 83–84% reported for *T. urticae* under similar metabolite exposure [11], highlighting a pronounced selectivity between pest and predator species—an essential requirement for integration into IPM programs.

From an applied perspective, the similar responses observed among *P. persimilis*, *N. californicus*, and *A. swirskii* facilitate the integration of *Trichoderma* into standardized biological control strategies. Although the observed adult mortality (up to 25%) is statistically significant, it falls within the “harmless” to “slightly harmful” categories defined by the International Organization for Biological Control (IOBC) for laboratory assays [29]. Moreover, the high egg hatchability suggests a strong capacity for population recovery, even when adult mortality occurs.

Nevertheless, the moderate impact on adults necessitates careful application strategies. Applications could be timed prior to predator release or aligned with periods of high pest density, where the benefits of rapid *T. urticae* suppression outweigh potential non-target effects. It should also be noted that laboratory bioassays represent a worst-case exposure scenario. Under greenhouse or field conditions, environmental factors such as UV radiation, temperature fluctuations, and plant architecture may reduce exposure and provide refugia for predatory mites, thereby mitigating adverse effects [30]. In addition, potential sublethal effects—such as changes in fecundity, longevity, or predation capacity—should be addressed in future studies to evaluate compatibility fully.

## 5. Conclusions

In conclusion, *Trichoderma* spore suspensions and secondary metabolites exhibited low to moderate non-target effects on phytoseiid predatory mites while maintaining high efficacy against *T. urticae*. This favorable selectivity profile supports the compatibility of *Trichoderma*-based applications with biological control agents used in IPM programs. Optimizing application timing and evaluating the sublethal and long-term effects of *Trichoderma* spp. on beneficial arthropods may further improve their role in integrated and sustainable mite management programs.

## Data Availability

All datasets generated and analyzed in this study are available from the corresponding author upon reasonable request.
